# Green Synthesized ZnO Nanoparticles Mediated by *Streptomyces plicatus*: Characterizations, Antimicrobial and Nematicidal Activities and Cytogenetic Effects

**DOI:** 10.3390/plants10091760

**Published:** 2021-08-25

**Authors:** Mohamed H. Kalaba, Saad A. Moghannem, Ahmad S. El-Hawary, Ahmed A. Radwan, Mohamed H. Sharaf, Abdelghany S. Shaban

**Affiliations:** Botany and Microbiology Department, Faculty of Science, Al-Azhar University, Cairo 11884, Egypt; Dr.m_kalaba@azhar.edu.eg (M.H.K.); Ahmad.hawary@azhar.edu.eg (A.S.E.-H.); Ahmedradwan@azhar.edu.eg (A.A.R.); mohamed.sharaf@azhar.edu.eg (M.H.S.); Abdelghanysobhy84@gmail.com or

**Keywords:** ZnO-NPs, *Streptomyces plicatus* MK-104, antimicrobial activity, plant pathogens, root-knot nematodes, cyto-genotoxicity, *Vicia faba*

## Abstract

Zinc oxide nanoparticles (ZnO-NPs) are regarded as one of the most promising kinds of materials in a variety of fields, including agriculture. Therefore, this study aimed to biosynthesize and characterize ZnO-NPs and evaluate their different biological activities. Seven isolates of actinomycetes were obtained and screened for ZnO-NPs synthesis. The isolate MK-104 was chosen and identified as the *Streptomyces plicatus* MK-104 strain. The biosynthesized ZnO-NPs exhibited an absorbance peak at 350 nm and were spherical in shape with an average size of 21.72 ± 4.27 nm under TEM. XRD and DLS methods confirmed these results. The biosynthesized ZnO-NPs demonstrated activity against plant pathogenic microbes such as *Erwinia amylovora*, *Aspergillus flavus*, *Aspergillus niger*, *Fusarium oxysporum*, *Fusarium moniliform* and *Alternaria alternata*, with MIC values ranging from 15.6 to 500 µg/mL. Furthermore, ZnO-NPs had a significant effect on *Meloidogyne incognita*, with death percentages of 88.2, 93.4 and 96.72% after 24, 48 and 72 h of exposure, respectively. *Vicia faba* seeds were treated with five concentrations of ZnO-NPs (12.5, 25, 50, 100 and 200 µg/mL). Low-moderate ZnO-NP concentrations (12.5–50 µg/mL) were shown to promote seed germination and seedling development, while the mitotic index (MI) decreased as the dosage of ZnO-NPs increased. Micronuclei (MNs) and the chromosomal abnormality index increased as well.

## 1. Introduction

Nanotechnology is currently generating a lot of excitement and it can be divided into three categories: physical, chemical and biogenic [1]. Even though all three approaches have been used to produce nanoparticles (NPs), the chemical and physical procedures are linked with environmental pollution, high temperatures, high pressures and expensive equipment [2,3]. Instead, biological approaches are increasingly being used for green nanoparticle synthesis [4,5]. They offer numerous advantages over other techniques, including being clean and cost-effective and generally having single-step protocols [6]. Furthermore, some of the most distinguishing characteristics of NPs produced biologically are their optical, photoelectrical and chemical characteristics, which allow them to be used for a wide range of medicinal, industrial and agricultural purposes [7,8,9,10,11]. Various biological materials, notably microorganisms, have demonstrated success in biologically manufacturing metal nanoparticles [12,13]. Bacteria, particularly actinobacteria, are an important group of microorganisms that could be used to create novel medicinal and industrial products, such as antimicrobials [14,15]. *Streptomyces* sp. belongs to the actinomycetes and its members are known for producing strong bioactive metabolites such as antibiotics. They are also thought to be suitable nanoparticle synthesizers since they can be produced both extracellularly and intracellularly through a broad range of actions [16,17]. Plant pathogens cause significant threats to economic and social stability all over the world and their prevalence is growing. Viruses, bacteria, fungi, nematodes and parasitic plants are among these plant pathogens [18]. Plant-parasitic nematodes, fungi and bacteria, particularly *Erwinia amylovora*, are some of the main elements that impact crop growth and development. As a result of extensive agricultural farming, parasites are multiplying at an alarming rate, resulting in losses in agricultural yield [19,20]. Root-knot nematodes, particularly *Meloidogyne* spp., are some of the greatest and most serious agricultural pests in Egypt, affecting a variety of field and vegetable crops, including tomato [21,22]. The management of these parasites is difficult and involves growing environmental concerns; thus, the continuous search for new nematode control strategies has recently accelerated. As a result, parasite management is critical to enhancing crop development. Artificial biocidal chemicals used to control pathogenic microorganisms can cause serious environmental hazards as well as pose a threat to mammalian health [23,24]. Engineered nanomaterials have the potential to interact with biomolecules and intracellular processes since many biological activities take place at the nanoscale level. From this point of view, ZnO-NPs have been of particular interest since they are thought to be non-toxic, safe and biocompatible. Additionally, ZnO-NPs have antimicrobial, optical, catalytic and electrical capabilities, as well as UV filtering qualities [25]. Over the last few years, the use of NPs in agriculture has been becoming more prevalent. There is growing interest in the use of nanoparticles to minimize reliance on chemical fertilizers for sustainable agricultural development and food security and fulfill the nutritional needs of the world’s fast-growing population [26]. The appropriate quantity of ZnO-NPs may have a beneficial impact on seed germination and seedling growth in a variety of plant species (including crops) [27,28,29]. Metallic zinc is an essential mineral for plant growth and development since it is included in several enzymatic and physiological activities [30]. Zinc is involved in the synthesis of protein, carbohydrates and nucleic acid, chlorophyll biosynthesis and energy production and in the metabolism of macromolecules, where it serves as an enzyme component, a catalyst or a structural cofactor [31]. Zinc enhances the seed germination rate, promotes rapid growth of radicals, influences water absorption and transport capacity and protects against the negative effects of temperature, drought and salt stress. Additionally, zinc plays a vital part in the formation of plant hormones like auxins and gibberellins [32,33,34]. Even though ZnO-NPs are among the most commonly manufactured nanoparticles in the world—after silver, carbon nanotube, titanium dioxide and gold NPs—only a few studies have been undertaken to examine the dual effects of biosynthesized ZnO-NPs on various plant pathogens and the cytogenic effects in plants [35]. In this study, we hypothesized that green production of ZnO-NPs may provide several benefits and properties. Therefore, the current work aimed to isolate and identify actinobacterial isolates capable of synthesizing ZnO-NPs extracellularly. The study aimed to assess the antibacterial and antinematodal efficacies of biosynthesized ZnO-NPs against different plant diseases, as well as the influence of biosynthesized ZnO-NPs on *Vicia faba* seed germination, shoot and root length and cytogenetic effects in vitro.

## 2. Results

### 2.1. Isolation and Screening of Actinobacteria for Synthesis of Zinc Oxide Nanoparticles

**Figure 1:**
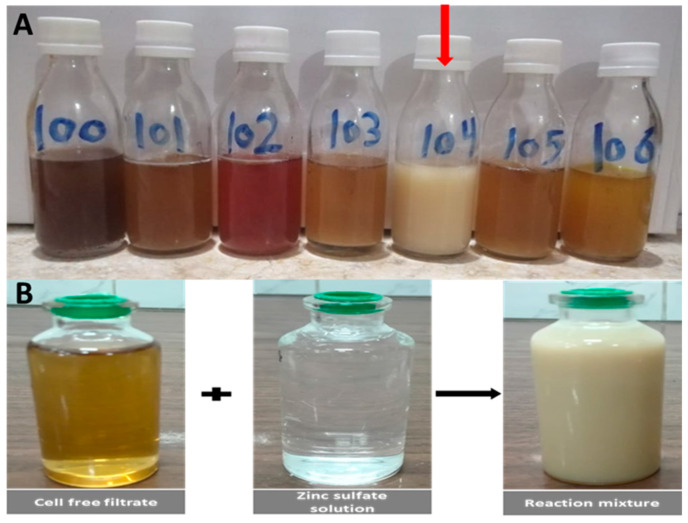
(**A**) Preliminary screening of ZnO-NP synthesis using different cultures of actinomycetes. (**B**) ZnO-NP formation from the CFF of isolate MK-104 using Zinc sulfate as a precursor.

Seven actinobacterial isolates coded as MK-100–106 were isolated from soil samples and screened for the biogenic synthesis of ZnO-NPs. Visual observation of the reaction mixture of cell-free filtrate (CFF) and zinc sulfate solutions was used for assessment in the preliminary screening for the synthesis of ZnO-NPs. According to this screening, MK-104 was the only isolate that changed the reaction mixture into a cloudy, -milky color, indicating biosynthesis of ZnO-NPs. In contrast, the other isolates did not show any milky cloudiness in the reaction mixture, indicating that they were unable to form ZnO-NPs, as shown in Figure 1A,B.

### 2.2. Characterization of Biogenic ZnO-NPs

**Figure 2:**
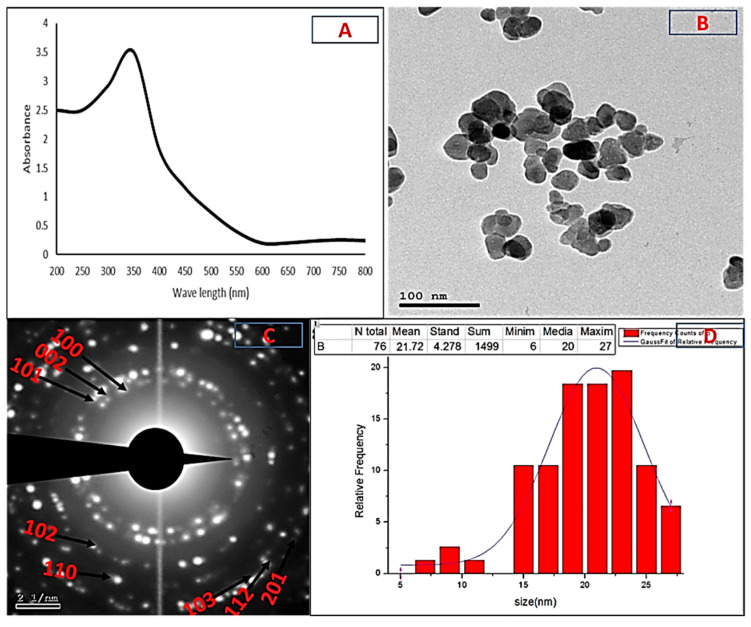
UV-visible absorption bands of ZnO-NPs synthesized from isolate MK-104 (**A**); transmission electron microscopic image of ZnO-NPs (**B**); SAED pattern of ZnO-NPs (**C**); chart of average size determination (**D**).

The UV-visible absorption spectra of the formed nanoparticles confirmed the previous visual observation. They showed a clear peak at 350 nm, a characteristic of ZnO-NPs, confirming their synthesis (Figure 2A). HR-TEM analysis showed that the ZnO-NPs were spherical in shape and had different particle sizes, with an average of 21.72 ± 4.27 nm. The HR-TEM image also showed that there were no aggregated forms (Figure 2B,C). Figure 2C shows the selected area electron diffraction (SAED) pattern, indicating that the biosynthesized ZnO-NPs were polycrystalline in nature.

**Figure 3:**
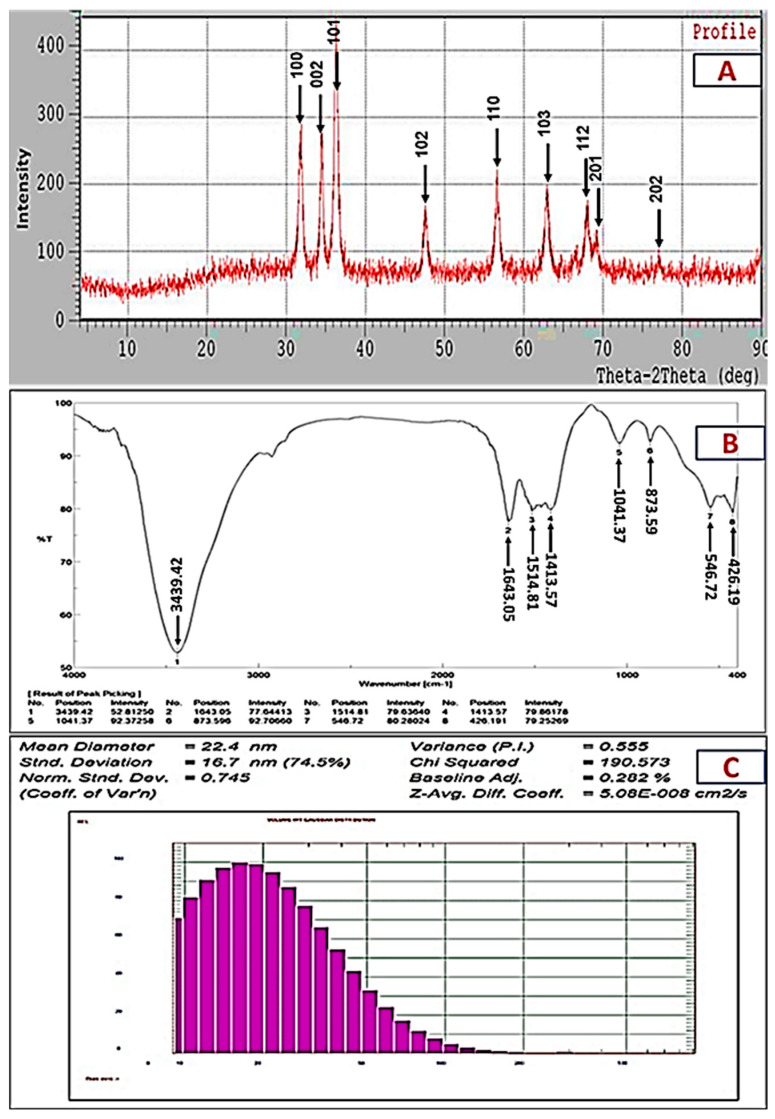
XRD pattern (**A**), FTIR spectrum (**B**) and particle size distribution (**C**) of ZnO-NPs formed from isolate MK-104.

Using the Debye–Scherrer equation, the XRD pattern of the ZnO-NPs revealed that they had a crystalline structure with an average size of 21.96 nm. The XRD profile also showed sharp and distinct peaks of 20 for 31.7°, 34.4°, 36.2°, 47.5°, 56.54°, 62.8°, 67.9°, 69° and 77°, which were indexed as the planes (100), (002), (101), (102), (110) (103), (112), (201) and (202) (Figure 3A). These peaks matched well with wurtzite ZnO from the Joint Committee on Power Diffraction (JCPD) standards, card number (36-1451). Thus, the XRD pattern revealed that ZnO-NPs with a fine hexagonal crystalline structure developed in close accordance with this reference model, and no other distinctive diffraction peaks were identified, suggesting that the bio-assisted NPs were free of additional phase impurities and had a high phase purity. Using an FTIR spectrophotometer, the biomolecules suspected to be responsible for the formation and efficient stability of the biosynthesized nanoparticles were identified. Figure 3B shows the IR spectra of the ZnO-NPs, with some vibration bands at 3439, 2910, 1643, 1514, 1413, 1041, 873, 546 and 426 cm^−^^1^. Generally, the intense wide peak at 3439 cm^−^^1^ is a characteristic of the hydroxyl functional group and the peak at 2910 cm^−1^ is the C-H stretching of the methylene group of proteins. The peaks at 1643 and 1514 cm^−^^1^ correspond to the carbonyl group and the ethylene group, respectively. The peak at 1413 cm^−1^ can be attributed to the protein amine II bands that were present in the sample. The C-N stretching vibrations were responsible for the observed band at 1041 cm^−1^. The peaks in the range from 400 to 900 cm^−^^1^ (873, 546 and 426 cm^−^^1^) can be attributed to the ZnO stretching mode, proving the creation and purity of the ZnO structure.

The DLS technique is a non-invasive technique for determining the size and distribution of nanoparticles scattered in liquid. The biosynthesized ZnO-NPs were observed to have a narrow size distribution, with various particle sizes and an average size of 22.4 nm, and a polydispersity index (PI) of 0.55 (Figure 3C). Using different characterization methods, it was concluded that the biosynthesized nanoparticles could be confirmed to be ZnO-NPs with a round crystalline form and an average size of 21.72–22.4 nm.

### 2.3. Identification of Isolate MK-104

**Figure 4:**
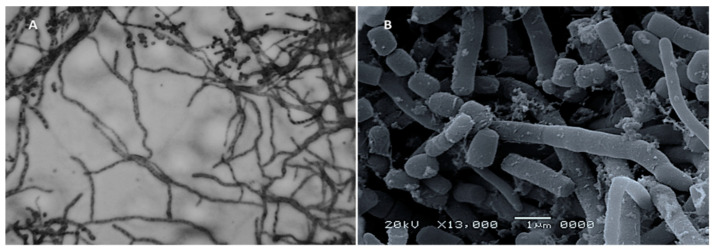
(**A**) Spore chain-bearing hyphae of the isolate MK-104 under light microscopy (×600); (**B**) Spore chain and spore morphology under scanning electron microscopy (×13,000).

Microscopic examination of the most potent actinobacterial isolate, MK-104, using the cover slip technique revealed that it formed a substrate and aerial hyphae with a long, straight to rectiflexible, branched aerial mycelium (Figure 4A). Scanning electron microscopy examination indicated that the aerial mycelia bore chains of cylinder-shaped spores that had smooth surfaces (Figure 4B).

The culture characteristics of the isolate MK-104 were recorded and included color observations for the sporulating aerial mycelium, substrate mycelium, diffusible soluble pigments and melanin pigment production after 14 days of incubation. The results for the culture characteristics are listed in Table 1.

Regarding the physiological characteristics, the isolate MK-104 could utilize and yield impressive growth on D-glucose-, L-rhamnose-, L-xylose-, D-mannitol-, mesoinositol-, L-arabinose- and D-fructose-supplemented medium, while sucrose and cellulose produced only moderate growth. It was also able to utilize different amino acids as a sole source of nitrogen, except for L-cysteine. The isolate MK-104 could survive at different temperatures from 20 to 50 °C and showed optimal growth between 30 and 40 °C. Growth of MK-104 was observed at a wide range of pH levels (5–9). NaCl tolerance test results showed that the isolate tolerated NaCl concentrations up to 7%, while it was suppressed at 8%. The growth was inhibited by sodium azide and phenol, while it succeeded on Czapek’s medium, and it tolerated crystal violet at 0.001% (*w/v*). The results for the physiological characteristics are recorded in Table 2.

Concerning the biochemical properties, the data listed in Table 3 explain the activity of MK-104 in the consumption of various substrates. The isolate had the ability to hydrolyze and break down lipids and starches, gelatin, tyrosine, urea, pectin, esculin and lecithin, while it was unable to hydrolyze casein. Motility and H2S production tests were negative but the citrate utilization test was positive.

**Figure 5:**
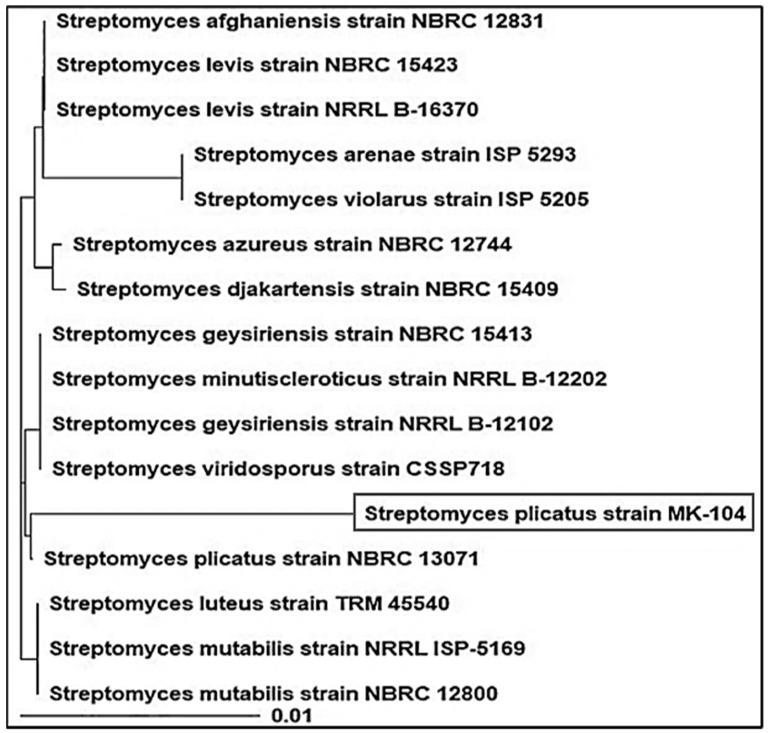
Phylogenetic tree of *Streptomyces plicatus* MK-104 strain as well as the most genetically similar *Streptomyces*-type strains.

In terms of molecular characterization, MK-104′s partial 16S rRNA gene sequence (1207 bp) was matched with the Streptomyces reference species in the GenBank database to confirm the identification at the genus taxon and it was shown to be strongly related to Streptomyces plicatus strain NBRC 13071, which had a 99% 16S rRNA gene similarity matrix (Figure 5). Accordingly, actinobacterial isolate MK-104 was identified as Streptomyces plicatus strain MK-104 and was placed in GenBank with accession number MN397912; the data for this isolate are available online.

### 2.4. Biological Activities of ZnO-NP Nanofluid

#### 2.4.1. Antimicrobial Activity

**Figure 6:**
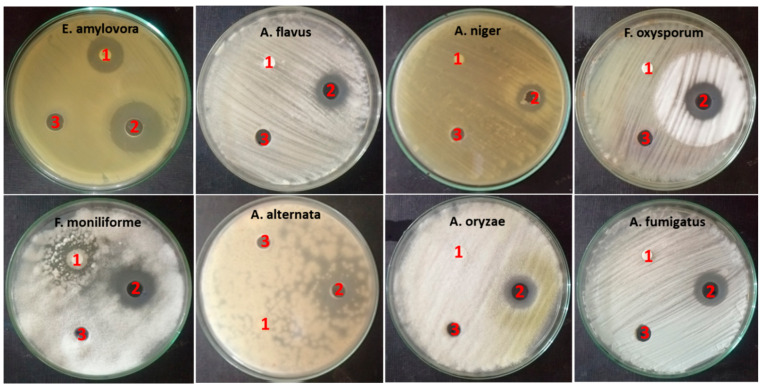
Antimicrobial activity of biosynthesized ZnO-NPs: (1) antibiotic controls, (2) ZnO-NP nanofluid and (3) base fluid.

ZnO-NP nanofluid exhibited action on all examined microorganisms, with inhibition zone diameters ranging from 15 to 26.6 mm. *E. amylovora* was the most sensitive strain to ZnO-NP nanofluid among the tested microorganisms, with an inhibition zone diameter of 26.6 mm. Furthermore, *Fusarium* fungi were considerably affected by ZnO-NP nanofluid, as the inhibition zones had diameters of 21 and 22 mm against *Fusarium oxysporum* and *Fusarium moniliform*, respectively, while *A. niger* was the least affected, with an inhibition zone diameter of 15 mm. In contrast, base fluid had no effect on any of the tested microorganisms, which proved the exclusive activity of ZnO-NPs. Antibiotic controls showed different results: trimethoprim/sulfamethoxazole demonstrated an inhibition zone diameter of 19.6 mm against *E. amylovora*, whereas fluconazole demonstrated an inhibition zone diameter of 17.6 mm against *Fusarium moniliform*. There were no effects on the rest of the tested fungi (Figure 6).

As the mechanisms of action of the nanoparticles on the bacteria were many and more varied than those on fungi, it might be concluded that bacteria require a smaller concentration of ZnO-NP nanofluid to be affected; however, it might alternatively be that fungi require greater concentrations to be inhibited. The MIC values listed in Table 4 support this theory. The lowest concentration of ZnO-NP nanofluid that inhibited *E. amylovora* was 15.6 g/mL, while the lowest concentration that inhibited the fungal growth began at 62.5 in the case of Fusarium oxysporum and increased to 500 g/mL in the case of *Alternaria alternata*.

#### 2.4.2. Nematicidal Activity of ZnO-NP Nanofluid

**Figure 7:**
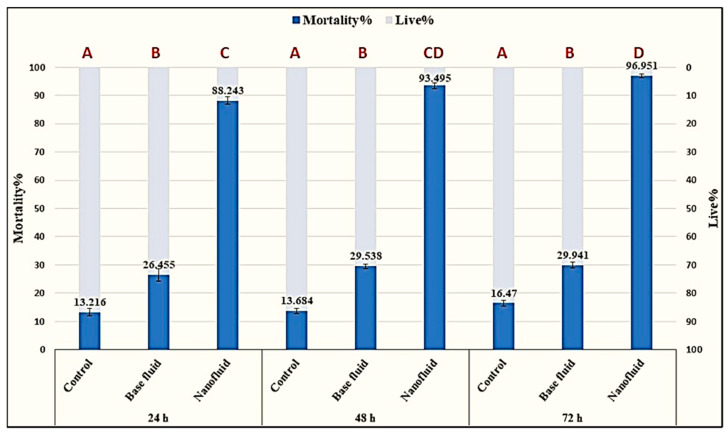
The percentages of deaths induced by ZnO-NPs after 24, 48 and 72 h of treatment; highly significant activity determined by the Tukey pairwise comparison test (*p* 0.05). letter “A” represent non sifnificance among control samples; ‘B” represent non significance among base fluid samples; “C & CD” represent the significance between ZnO-NPs at time 24, 48 and 72 h. the highly significant activity was denoted with the letter “D”.

**Figure 8:**
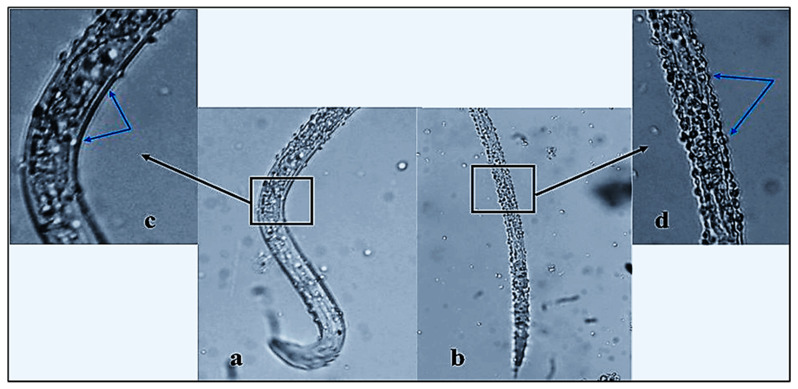
(**a**) The mobility (winding shape) of J_2_s indicates the viability and (**b**) the immobility (straight shape) indicates the death of J_2_s (300×). (**c**) Smooth surface of vital J_2_s cuticle and (**d**) serrated cuticle of dead J_2_s as a result of ZnO-NP nanofluid exposure for 72 h.

The number of living M. incognita J_2_s decreased significantly after 24 h of ZnO-NP exposure in vitro and mortality reached 96.9% after 72 h. According to bioassay results, ZnO-NP nanofluid significantly affected J_2_s compared to control and base fluid (*p*  ≤  0.05). The number of living M. incognita J_2_s decreased dramatically after only 24 h of contact with ZnO-NP nanofluid, with a mortality percentage of 88.2%. Further exposure for another 24 h showed a non-significant increase in the death rate that reached 93.4% (indicated by “CD”; Figure 7). Finally, the effect after 72 h of exposure achieved a climax in mortality, with 96.9% recording more significant nematicidal activity when compared to the first day of exposure. In addition to the remarkable immobility of dead J_2_s, the effects of the ZnO-NP nanofluid included deformation of the nematode cuticle, which was obvious under a light microscope with a high-power lens (Figure 8b,d). In contrast, the worm body was in good condition, with a smooth surface and distinct lateral striae and resting in a curved position (Figure 8a,c).

### 2.5. Impact of ZnO-NPs on Germination and Seedling Vigor

**Figure 9:**
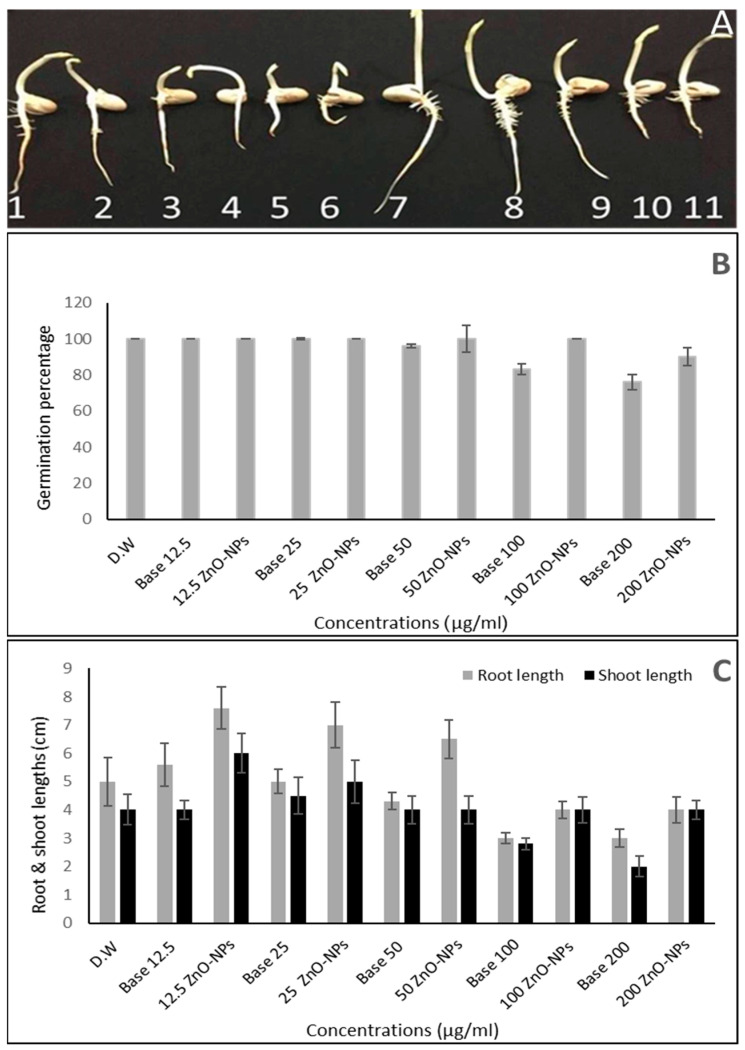
Effects of various ZnO-NP concentrations on Vicia faba. (**A**) The treated plants’ morphological characteristics at all concentrations—1: D.W; 2–6: base fluid dilutions; 7–11: ZnO-NP nanofluid concentrations; (**B**) percentages of of seed germination; (**C**) shoot length and root length (cm).

The influence of ZnO-NP nanofluid concentrations and base fluid dilutions on the percentage of germination in the various treatments over five days is presented in Figure 9A,B. Seed germination began after one day, with a percentage ranging from 26.6 under control conditions to 20% with 12.5 µg/mL ZnO-NP nanofluid. Seed germination began on the first day as well, but with a low percentage (less than 20% in all the treatments except at 200 µg/mL concentrations, even in the nanofluid and in the base fluid, where it was registered as zero). After 5 days of imperfect treatments, the highest percentage of germination (100%) was achieved, and the lowest percentage of germination was reported with dilutions of 200 and 100 base fluid and 100 µg/mL nanofluid (76 ± 4, 83 ± 3 and 90 ± 5, respectively).

ZnO-NP treatments influenced both root and shoot lengths (Figure 9A,C). Root and shoot elongation was stimulated by low-moderate levels of ZnO-NPs, reaching a height of 7.6 ± 0.8 and 6 ± 0.7 cm, respectively, with 12.5 µg/mL of ZnO-NPs, following which the roots steadily decreased at higher concentrations of 100 and 200 µg/mL to 4 ± 0.3 and 4 ± 0.45 cm, respectively.

In the case of base fluid, there was no substantial difference in root lengths between low and moderate levels compared to control, but they decreased drastically at high levels and registered the shortest lengths among all treatments at 100 and 200 base fluid dilutions (2.8 ± 0.2 and 2 ± 0.33 cm, respectively).

### 2.6. Effect of ZnO-NPs on Mitotic Index and Chromosomal Aberration

**Figure 10:**
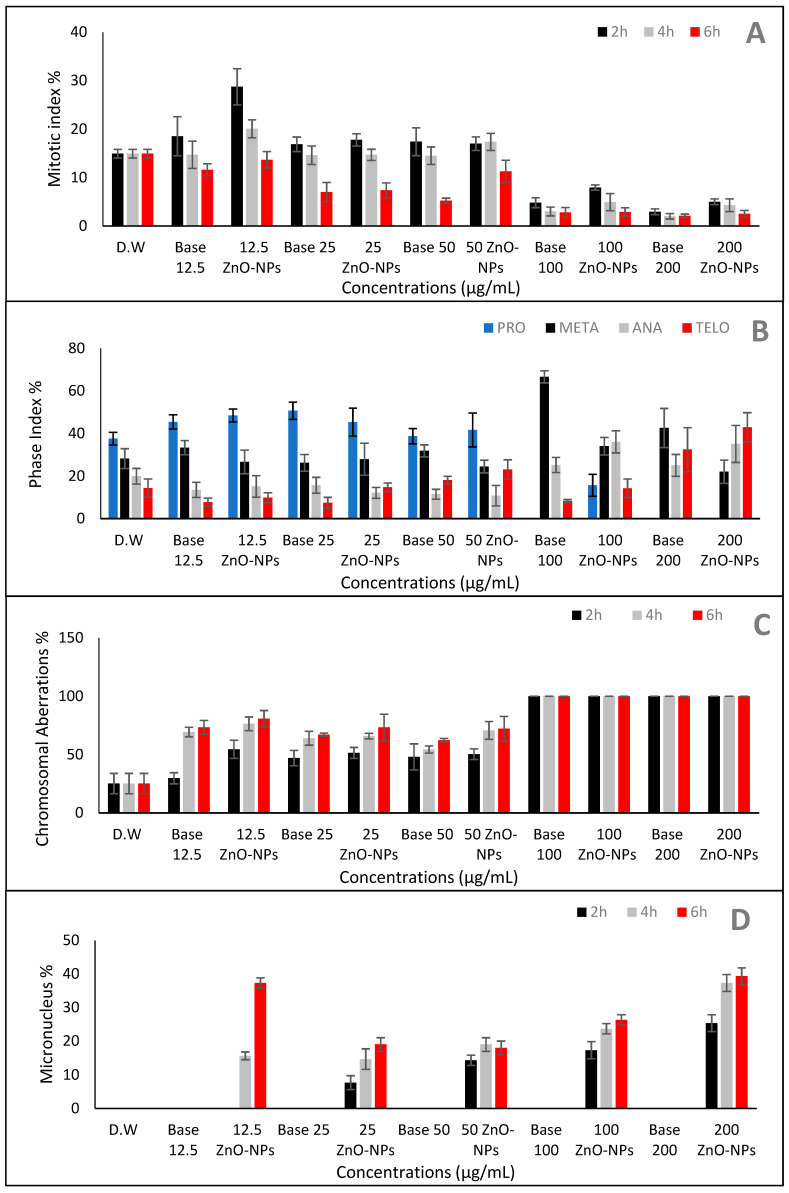
Cytotoxic effect of ZnO nanoparticles on root meristems of *Vicia faba*: (**A**) mitotic index (%), (**B**) phase index (%), (**C**) chromosome aberrations (%), (**D**) micronucleus (%).

The cytotoxic and genotoxic capabilities of ZnO-NP nanofluid against *Vicia faba* seeds was estimated by observing cytological parameters such as the mitotic index and the number and percentage of chromosomal abnormalities. The percentage of dividing cells increased dramatically prior to the addition of 100 µg/mL ZnO-NP nanofluid, after which the value dropped significantly. The value of the mitotic index was reduced by more than half at dose concentrations of 100 and 200 µg/mL of ZnO-NP nanofluid and for the 100 and 200 dilutions of base fluid as compared to the control at the low time duration (7.9 ± 0.55, 5 ± 0.60, 4.8 ± 1.01 and 2.9 ± 0.60, respectively). The highest percentage for the mitotic index (28.7 ± 3.75) was recorded for the 12.5 µg/mL ZnO-NP nanofluid treatment at 2 h, while the lowest was found for the 4 h treatment with the 200 base fluid dilution (2 ± 0.55; Table 5 and Figure 10A).

The mitotic index (MI) reduction was found to be statistically highly significant for all treatments at 6 h, with minimum values of 2.1 ± 36 and 2.5 ± 0.7% for the 200 base fluid dilution and 200 g/mL ZnO-NP nanofluid, respectively). It might be deduced that there was a direct link between increased exposure time, ZnO-NP nanofluid concentration and decreased mitotic activity. Before the application of the 100 base fluid dilution, the prophase was the most common mitotic stage for all exposure concentrations measured (Table 5). However, increasing the nanoparticle dose resulted in a significant increase in the phase index values for certain divisional levels, such as the anaphase and telophase, which peaked at 100 and 200 µg/mL ZnO-NP nanofluid, respectively (Table 5 and Figure 10B).

The current study found that multiple chromosomal defects were induced at all stages of the cell cycle, which also led to alterations in the mitosis stages and nuclear membrane damage. The rate of chromosomal aberration increased as the exposure period or ZnO-NP nanofluid concentration increased, as shown in Figure 10C. For all concentrations tested, the chromosomal aberration percentages were found to be higher. Aberrant chromosomes were visualized with their maximum percentage abnormality at higher nanoparticle dosages (100–200 µg/mL) and 100 and 200 base fluid dilutions (100%). Even at 200 µg/mL of ZnO-NP nanofluid, the influence on the MI was not inhibited. When the ZnO-NP nanofluid at 200 µg/mL was excessively toxic, it could cause cell death and interfere with cell scoring for ZnO-NP-induced aberrations.

**Figure 11:**
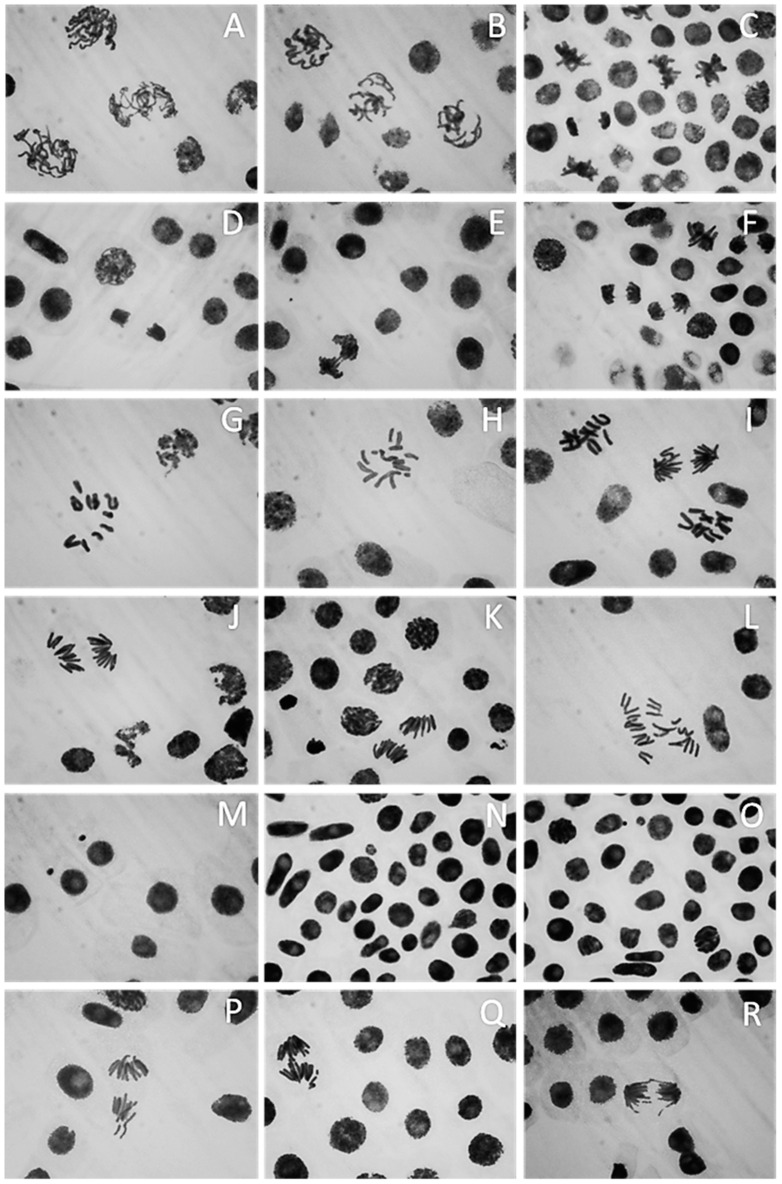
Different treatments of ZnO nanoparticles caused anomalies in *Vicia faba* root meristems: (**A**,**B**) irregular prophase; (**C**–**F**) sticky chromosome (prophase, anaphase and telophase with bridges, respectively); (**G**–**I**) C-metaphase; (**J**,**K**) disturbed anaphase, (**L**) C-anaphase; (**M**–**O**) micronucleus at interphase; (**P**–**R**) chromosome breakage and forward at anaphase.

Figure 11 shows that the ZnO-NP nanofluid caused various forms of abnormal mitotic cells in the roots of the faba bean. Stickiness, chromosome bridges, irregular prophases, C-metaphases, C-anaphases, ring chromosomes, bridges and micronuclei were the most common abnormal cells. Disturbed condensation was observed in the prophase with DNA and proteins condensed in an unusual fashion. The formation of the metaphase plate in the middle of the cell was not in an aligned manner and the chromosomes were not correctly paired, which resulted in anaphase disruption, causing chromosome movement towards each pole of the cell to be hampered (Figure 11G,H). Chromosomal breakage, disruption and phase delay were also observed during the anaphase stage. Stickiness and condensation of chromosomes were detected during the metaphase, anaphase and telophase stages (Figure 11C–F). Both the negative and vehicle controls had non-micronucleated cells. The highest occurrence of MNs was seen at 200 µg/mL after 6 h of treatment and the lowest at 25 µg/mL after 2 h in the case of the ZnO-NP nanofluid concentrations (Figure 10D and Figure 11M–O). For all measured ZnO-NP nanofluid concentrations, the percentage of micronucleated cells changed with the dose and time-dependently.

## 3. Discussion

Several studies have been undertaken in the field of ZnO-NP synthesis using different biological materials and the authors stated that the synthesis of ZnO-NPs was observed as a white, cloudy haziness in the solution of the reaction mixture, which became deposited at the bottom of the flasks [36,37,38]. As the metabolites in each isolate’s CFF may be varied, the results can change, as was the case in this investigation. Thus, the isolates that were able to form nanoparticles were determined by the presence of cloudiness in the reaction mixture. According to our results from the preliminary screening, MK-104 synthesized ZnO-NPs. As each microbe has a particular metabolic process and enzyme activity, not all microbes can synthesize NPs. In this sense, choosing the right microorganisms (independently of their enzyme activity or metabolic pathways) is critical for the formation of NPs [39]. Previous research on biosynthesis of ZnO-NPs utilizing microbes and plants reported absorption peaks at the same range. For example, Yusof et al. (2020) formed ZnO-NPs via bacterial cells and cell-free filtrate of *Lactobacillus plantarum* TA4, and the UV-vis absorption spectrum analysis gave absorption peaks at 349 and 351 nm, respectively [38]. Ehsan and Sajjad (2017) synthesized ZnO-NPs with a 360 nm absorption band using zinc sulfate as a precursor and *Ficus carica* leaf extract as a reactant [37]. The TEM and SAED pictures verified the formation of crystalline metal nanostructures and gave more insight into the spherical shape and size features of the metal nanoparticles [40]. The current study’s TEM picture showed that the produced ZnO-NPs were effectively dispersed, indicating that the CFF of *Streptomyces plicatus* strain MK-104 had good capping and stability capabilities. As the size of the ZnO-NPs produced in this work was small (21.72 nm), we anticipate that they can be used in a variety of biotechnological applications. The average crystalline size was estimated using the Debye–Scherrer equation and found to be 21.96 nm after XRD examination. The XRD profile of the investigated ZnO-NPs showed significant intensity and small width for the diffraction peaks, indicating the good crystallinity of the final product. There were no additional diffracted peaks of other phases detected, indicating the phase purity of the ZnO-NP powder. In addition to this, the peaks found in this study were in good accordance with the spherical and hexagonal wurtzite structure of ZnO-NPs, as confirmed by comparison with the Joint Committee on Powder Diffraction (JCPD) standards, card number (36-1451), and prior studies [41,42,43]. Drawing on the results of the FTIR analysis of the ZnO-NPs, the presence of the hydroxyl functional group, carbonyl group and ethylene group may indicate the presence of carbohydrates and/or alcohols, ketone and/or quinones and alkene compounds, respectively. In addition, the presence of C-H stretching of methylene and N-H and C-N stretching might also indicate the presence of protein compounds. Kapoor et al. (2021) reported that the biosynthesis of nanoparticles might occur through protein compounds, mainly via reducing enzymes, located on the cell membrane of microbial cells or released to the growth medium as extracellular enzymes [44]. In some cases, the non-enzymatically mediated synthesis depends on certain organic functional groups present on the microbial cell wall, which facilitate the reduction of metal ions [39]. Our findings revealed the presence of protein and amide I and II bands, which may have a role in the formation and capping of the metals [45]. In addition, the metal oxides also had absorption peaks less than 1200 cm^−1^, which were caused by interatomic vibrations and represented the fingerprint region of the zinc oxide nanoparticles [41,46]. The polydispersity index (PI) generated from the DLS analysis represents the second moment of the nanoparticle population’s size distribution. Monodispersed particles are represented by PI ranges from 0.01 to 0.5 or 0.7. Polydispersity index values > 0.7 were detected in samples that had extremely extensive size distributions [47]. The obtained results revealed that the PI value of the ZnO-NPs produced by the *Streptomyces plicatus* strain MK-104 was 0.55, indicating that the colloidal solution was homogeneous and nearly monodispersed. The size of the ZnO-NPs (average size of 22.4 nm) was slightly greater in the DLS analysis compared to that determined by other methods, such as TEM and XRD. The coating agent that capped and stabilized the surfaces of the NPs was responsible for this increase. Furthermore, the non-homogeneous NP distribution in the colloidal solution might explain the higher size in the DLS analysis [48].

According to Williams et al. (1989) and Hensyl (1994), the morphological, physiological and biochemical features of the actinobacterial isolate MK-104 underline that this isolate should be classified at the genus level as *Streptomyces* sp. [49,50]. With the aid of genetic identification and alignment of the 16S rRNA gene sequence on GenBank to compare it with the most similar sequences, MK-104 was identified at the species level as *Streptomyces plicatus* strain MK-104. To our knowledge, *Streptomyces plicatus* strain MK-104 has never been employed for ZnO-NP production.

ZnO-NPs previously synthesized with biological enzymatic approaches showed outstanding antibacterial and antifungal properties [51,52]. The antibacterial action of ZnO-NPs is thought to be related to the generation of reactive oxygen species (ROS), which causes oxidative stress and cell death [53]. The antibacterial action of ZnO-NPs might be attributed to the NPs’ electrostatic attachment to the microbial membrane, which changes its composition, destroys cell integrity and, subsequently, leads to leakage of intracellular contents and cell death. The antibacterial activity of ZnO-NPs may also be due to the NPs’ electrostatic attachment to the microbial cell membrane, which alters the composition of the cell membrane and damages the cell integrity, resulting in intracellular content leakage and cell death [54,55]. The treatment of fungi with ZnO-NPs significantly reduced conidial formation and deformed the conidiophores of *Penicillium expansum* and *Botrytis cinerea*, displaying antifungal properties, according to He et al. (2011) [56]. When particles are reduced in size from micrometres to nanometers, their characteristics can drastically alter. For example, electrical conductivity, hardness, active surface area, chemical reactivity and biological activity have all been known to change. Palanikumar et al. (2014) investigated the antibacterial activity of ZnO-NPs of various sizes and concentrations. It was discovered that the greatest concentration of ZnO-NPs (200 µg/mL^−1^) and the smallest particle size (15 nm) significantly inhibited microbial growth. As a result, it was determined that the inhibitory effectiveness of ZnO-NPs is highly dependent on the concentration and size used [57]. This may be attributed to the fact that nanoparticles’ reduced size makes it easier for them to pass through the microbial cell membrane, inhibiting cell growth and promoting bacterial cell death [58].

Compared to the first day of exposure, the impact of the ZnO-NPs attained a peak in mortality after 72 h of exposure at 96.9% and demonstrated substantial additional nematicidal activity. A previous study assessed the effect of biosynthesized ZnO-NPs on root-knot nematodes (RKNs) and reported that, under in vitro conditions, ZnO-NPs caused 72.08% mortality after 72 h [59]. In addition, the abnormal appearance of *M. incognita* J_2_s under the light microscope suggests that the ZnO-NP nanofluid might have strongly affected the structural protein of the juveniles’ bodies, which might have been correlated with their deaths [60]. Low concentrations of ZnO-NPs (especially 12.5, 25 and 50 µg/mL) promoted *Vicia faba* seed germination and seedling development, but higher concentrations (100 and 200 µg/mL) induced phytotoxicity. These results are consistent with those of Youssef and Elamawi (2020) [61]. In addition, Upadhyaya et al. (2017) found that ZnO-NPs increased rice seed germination, which is consistent with our findings [62]. Raskar and Laware (2014) examined the effects of ZnO-NPs with a diameter of 20 nm and concentrations of 0, 10, 20, 30 and 40 mg/L on onion root length (*Allium cepa* L.) [63].

**Figure 12:**
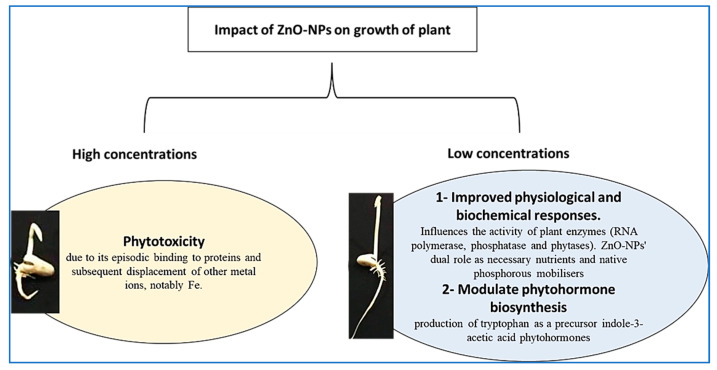
Schematic diagram of the effects of different concentrations of ZnO-NPs on plant growth.

At lower concentrations, ZnO-NPs increased root length, but at higher concentrations, they decreased it. The observed increases in shoot and root lengths in response to low ZnO-NP concentrations might represent a nutritional impact of the ZnO-NPs, whereby they influence the activity of RNA polymerases and other plant enzymes, acting as cofactors in a variety of metabolic and physiological cycles (Figure 12). Zinc oxide NPs in the rhizosphere boost the activity of phosphorous mobilization enzymes, such as phosphatase and phytases, increasing the amount of phosphorous accessible to plants [64,65]. As a result, the improved physiological and biochemical responses during seed germination and early seedling growth are consistent with ZnO-NPs’ dual role as necessary nutrients and native phosphorous mobilisers [66].

However, because of their occasional interaction with proteins and the resulting displacement of other metal ions, including Fe, they are toxic when accumulated in large amounts [67]. The MI is a decent cytotoxicity predictor [68]. According to our findings, the exposure of *Vicia faba* roots to ZnO-NPs may have a cytological impact. In *Allium cepa* and *Allium sativum* L., there was a dose-dependent inhibition of the mitotic index, indicating that ZnO-NPs had cytotoxic potential [69,70]. We agree with the report by Youssef and Elamawi (2020) in which different ZnO-NP treatments caused different increases in MI values in the *V. faba* root cell, with the highest value observed for the smaller concentration (10 mg/L) [61]. We also agree with an earlier study [71,72,73] that discovered a reduction in MI rates in the root tips of *A. cepa* and *V. faba* when Zn or ZnO-NP concentrations and exposure durations increased. The improved root and shoot growth reported for 12.5, 25 and 50 µg/mL treatments reflected the increased MI with low ZnO-NP concentrations. The reduction in mitotic activity, particularly at higher concentrations and for longer periods of time, most generally reflects a mitodepressive influence of ZnO-NPs, which may prevent some cells from entering the prophase and block the mitotic cycle during the interphase, inhibiting DNA/protein synthesis [74].

Sticky chromosomes were thought to be corroborated by the high DNA fragmentation increase observed [75]. Stickiness, according to Nwakanma and Okoli (2010), is a sign of high toxicity and leads to inappropriate protein–protein interaction [76]. This reflects the toxic effect of ZnO-NPs, which is usually irreversible and results in cell death. Stickiness and other chromosomal aberrations identified in this study could be attributable to ZnO-NPs binding to DNA and proteins, producing hazardous alterations in their physical and chemical properties; nucleus chromatin condensation; or the development of inter- and intra-chromatid crosslinks. Bridges, C-metaphases and illness at the anaphase and telophase were also observed in the meristematic cells of *Vicia faba* subjected to the ZnO-NPs. These abnormalities indicated structural chromosomal rearrangements and a probable clastogenic character for the ZnO-NPs. The aneugenic action of Zn compounds was also demonstrated by the high values of C-metaphases, which was likely due to the perturbation of calmodulin, a small Ca^2+^-binding protein involved in chromosome movement via microtubule polymerization/depolymerization control [77]. The frequency of MNs in *V. faba* root cells may indicate that ZnO-NPs have genotoxic and cytotoxic properties. Ghosh et al. (2016) found similar genotoxic effects of ZnO-NPs in *A. cepa* and *V. faba* [73]. The micronucleus test was previously used to determine the toxicity of ZnO-NP-polluted soil for *V. faba* root-tip cells [78]. Micronuclei were found intermittently in all ZnO-NP treatments; these could have been acentric fragments (clastogenic reaction) or the product of mitotic spindle dysfunction (aneugenic response) [79]. The genotoxic potential of zinc is supported by the very wide ranges in the rates of metaphase abnormalities and ana-telophase chromosome aberrations. The high number of chromosome aberrations suggests that they interfere with nucleic acids and that Zn has clastogenic potential, while the perturbations implying the mitotic spindle demonstrate that it has an aneugenic effect. Venkatachalam et al. (2017) demonstrated the importance of ZnO-NPs as a plant growth stimulant and observed that plants treated with a variety of nanoparticle concentrations had higher percentages of carotenoids, biomass, chlorophyll a, total soluble proteins, superoxide dismutase (SOD) and peroxidase (POX), but lower percentages of catalase (CAT) and malondialdehyde. This may have occurred as a result of increased antioxidant defense enzyme activity, which regulates isoenzyme expression patterns on the one hand and reduces reactive oxygen species (ROS) on the other. As a result, ZnO-NPs integrated in biomolecules are a promising choice for agricultural applications [80]. On the other hand, zinc nanoparticles may be toxic. Two hypotheses can be tested to identify their toxicity: the first hypothesis relates to the chemical toxicity based on the chemical composition and concentration, while the second concerns the stress generated by the size, shape and surface of the ZnO-NPs. Both parameters have a substantial impact on plants’ cell responses [81].

## 4. Materials and Methods

### 4.1. Isolation and Screening of Actinobacteria for the Biosynthesis of ZnO-NPs

Five soil samples were collected from Ekhtab, Aga, Aldakahleia governorate, Egypt (30°49′21.94″ N, 31°18′40.62″ E). Actinobacteria isolates were obtained using the soil dilution plate technique and purified as described by Sineva and Terekhova (2015) [82]. These isolates were screened for the biosynthesis of ZnO-NPs according to the method reported by Ashajyothi et al. (2016) [83]. Briefly, fresh seed cultures of actinobacterial isolates were prepared by inoculating two loopfuls of 7 day old culture in a 250 mL conical flask that contained 50 mL of starch nitrate broth medium (the pH was adjusted to 7.2 before sterilization using 1 N NaOH or 1 N HCl, (analytical grade, Adwa, Egypt)) and placing it in a shaker incubator at 150 rpm, 30 °C, for 48 h. A 100 mL flask containing 20 mL sterile starch nitrate broth medium was inoculated with an 8% (*v/v*) seed culture of each actinobacteria isolate separately and incubated in a shaker incubator at 150 rpm, 30 °C, for 10–14 days. The culture of actinobacterial isolates was filtered through a cotton layer and then subjected to centrifugation at 10,000× *g* rpm for 15 min to obtain cell-free filtrate (CFF). Each isolate’s CFF was introduced individually to reaction containers that contained 1.5% (*w/v*) zinc sulfate solution (ZnSO4·7H_2_O, HiMedia, Mumbai, India), which was employed as a zinc precursor at a 1:1 (*v/v*) ratio. The CFF and metal solution were maintained separately during the experiment as a control. The pH of the reaction mixtures was adjusted to 7 and then they were placed in a rotary shaker at 37 °C for 72 h and centrifuged at 120 rpm in dark conditions. Biosynthesized zinc oxide nanoparticles were indicated by visual observation of the reaction mixture changing in color to a cloudy or milky solution.

### 4.2. Purification of Biosynthesized ZnO-NPs

To acquire the precipitate, the cloud solution produced by the reaction of zinc sulfate with the filtrate of the chosen isolate was centrifuged at 10,000× *g* rpm for 15 min. To eliminate the remaining biological molecules, the metal nanoparticles in the form of pellets were washed three times with sterile deionized water and centrifuged at 5000× *g* rpm for 15 min after each washing. The purified metal nanoparticles were resuspended in the minimum volume of sterile deionized water and poured into a pre-weighed glass petri dish, then dried in an oven at 90 °C until a stable weight was obtained [84]. The metal nanoparticle powder was then resuspended in 10 mL deionized water and maintained on a sonicator (Anonkia, Shenzhen, China) to prevent ion aggregation before being subjected to characterization.

### 4.3. Characterization of Biosynthesized ZnO-NPs

The UV-Vis spectroscopy study was performed using a Unico 2100 UV-visible Spectrophotometer (Ridge Road, Suite E Dayton, NJ, USA)) at wavelengths ranging from 200 to 800 nm to confirm the production of ZnO-NPs, and deionized water was utilized as a blank. The UV-visible spectrum’s strong peak verified the formation of ZnO-NPs at a maximum surface plasmon resonance peak. [45]. High-resolution transmission electron microscopy (HR-TEM) was performed to determine the size and shape of the biosynthesized ZnO-NPs. In brief, the powder of the ZnO-NPs was mixed in absolute ethyl alcohol and the ZnO-NP solution was doped on a carbon-coated copper grid and air-dried, then examined using a JEM-2100F (JEOL, tetchikawa, Tokyo, Japan) at the National Research Center (NRC), Giza, Egypt. The particle size distribution of the biosynthesized ZnO-NPs was analyzed using ImageJ software origin 8 to obtain the average size from at least 100 measured particles [85]. The crystalline structure of the biosynthesized nanoparticles was characterized by X-ray diffraction [86]. The average size of the biosynthesized nanoparticles was calculated from XRD analysis according to the Debye–Scherrer equation:

D = Kλ/β cos θ

where D is the crystalline size, K is the shape constant, λ is the wavelength of the X-ray, β is the full maxima half-width and θ is the diffraction angle. This analysis was undertaken using a Shimadzu apparatus (Shimadzu Scientific Instruments (SSI), Kyoto, Japan) with a nickel filter and Cu-Ka target. Fourier-transform infrared (FTIR) analysis of biosynthesized nanoparticles was carried out using an Agilent system Cary 630 FTIR Spectrometer (Creek Blvd, Santa Clara, CA, USA). The powder of the biosynthesized nanoparticles was placed in a micro-cup with a 2 mm internal diameter and this was inserted in the FTIR set, at 26 °C ± 1 °C, and scanned with infrared in the 4000–400 cm^−1^ range. The resulting spectral data were compared to a reference chart to determine the functional groups that existed in the sample [87]. The particle size distribution of the biosynthesized nanoparticles was measured using a Malvern Zetasizer Instrument. Dynamic light scattering (DLS) measurements were performed between 10 and 1000 nm and the collected data were examined using the Zetasizer software version 7.11 [88].

### 4.4. Identification of the Selected Actinobacterial Isolate

The selected isolate was characterized morphologically, physiologically and biochemically. The morphological investigation was performed microscopically with a light microscope (Optika, Via Rigla, Ponteranica, Italy) using the coverslip technique [41] and with scanning electron microscopy (JEOL Technics Ltd., Tokyo, Japan) according to Moghannem et al. (2017) [89]. Macroscopically, the culture characteristics were described on various International Streptomyces Project (ISP) media after cultivation for 14 days at 28 °C. The utilization of various carbon and nitrogen sources by the selected actinobacterial isolate was studied at 28 °C for 15 days in ISP-9 media. The pH and temperature ranges of growth were also investigated on starch nitrate medium [90]. The ability of the actinobacterial isolate to grow in the absence and presence of NaCl at varied concentrations (0, 1, 2, 3, 4, 5, 6, 7, and 8% (*w/v*)) was examined on starch nitrate medium and the results were recorded after cultivation for 14 days at 28 °C. The growth on Czapek’s medium and tolerance to toxic substances, such as sodium azide 0.01% (*w/v*), phenol 0.1% (*w/v*) and crystal violet 0.001% (*w/v*), were assessed according to Shirling and Gottlieb (1968) [91]. Biochemical tests, including tests for lipid, starch, gelatin and casein hydrolysis; tyrosine, urea, pectin, esculin and lecithin degradation; motility; citrate utilization; and H_2_S production, were performed [92]. All chemicals and the media used for identification were purchased from Merck (Germany). Using the findings of the aforementioned investigations, the actinobacterial isolate was identified at the genus level using Bergey’s Manual of Determinative Bacteriology [93]. Further identification was performed through 16S rRNA gene analysis according to Atta et al. (2011) [94].

### 4.5. Preparation of ZnO-NP Nanofluid

To obtain stable and homogenous suspension of ZnO-NPs for assessment of biological activities, nanofluid was prepared according to Saliani et al. (2015) with some modifications [43]. In brief, 2.5 gm of ZnO-NP powder was dispersed in 25 mL of glycerol (Merck, Darmstadt, Germany) and mixed using a high mechanical shear mixer at 12,000 rpm (Heidolph Diax 600, Taufkirchen, Germany) for 30 min. The stabilization of the ZnO-NP–glycerol suspension was improved by adding ammonium citrate (Merck, Darmstadt, Germany) as a dispersant with a weight ratio of 1:1 compared to the ZnO-NPs. Ammonium citrate was dissolved in 25 mL of double-distilled water and added to the ZnO-NPs–glycerol suspension, which was then mixed for one hour at the previous speed to produce well-dispersed nanofluids with a final concentration of 50 mg/mL. Base fluid was prepared similarly to the previous manner, with the same components, but without adding ZnO-NP as a control.

### 4.6. Biological Activities of ZnO-NP Nanofluid

#### 4.6.1. Antimicrobial Activity and Determination of the Minimum Inhibitory Concentration (MIC) of ZnO-NP Nanofluid

Antimicrobial activity of ZnO-NP nanofluid was evaluated against the plant pathogenic bacterial strain *Erwinia amylovora* and five plant pathogenic fungal strains (*Aspergillus flavus*, *Aspergillus niger*, *Fusarium oxysporum*, *Fusarium moniliform* and *Alternaria alternata*), in addition to non-plant pathogens *Aspergillus oryzae* and *Aspergillus fumigatus*. These plant pathogenic cultures were obtained from the Mycology Laboratory, Microbiology Department, Faculty of Science, Al-Azhar University, Cairo, Egypt. For the preparation of the seed cultures of these microorganisms, a loopful of two-day- or seven-day-old culture for bacteria and fungi, respectively, was inoculated in conical flasks of 100 mL, each containing 20 mL of tryptic soy broth and potato dextrose broth media (Merck, Darmstadt, Germany), respectively. The inoculated flasks were incubated for 24 and 48 h at 37 °C and 28 °C for bacteria and fungi, respectively. The antimicrobial activity of the ZnO-NP nanofluid was determined by the agar well diffusion method according to Sharaf et al. (2021) and Pulit-Prociak et al. (2021) with some modifications [95,96]. Muller Hinton agar and potato dextrose agar (Merck, Darmstadt, Germany) plates were inoculated with 100 µL of the prepared bacterial (10^6^ CFU/mL) and fungal (1.5–5.0 × 10^7^ CFU/mL) seed cultures respectively and distributed with a sterile cotton swab on the surface of the prepared media. With a sterilized cork borer, agar cups (8 mm in diameter) were cut from the pre-inoculated plates. One hundred microliters of ZnO-NP nanofluid at a concentration of 1000 µg/mL was transferred to the agar cup. One hundred microliters of the base fluid was also transferred into another cup to be used as vehicle control. In addition, the antibiotic control used for the bacteria was trimethoprim/sulfamethoxazole (25 µg/mL) and the antifungal control used for the fungi was fluconazole (25 µg/mL) (Bioanalyse, Ankara, Turkey). All plates were kept at 4 °C for 2 h to allow the investigated compounds to diffuse. The plates were then incubated for 24 h at 37 °C for bacteria and 72–96 h at 28 °C for fungi. After incubation, the diameters of the zones of inhibition were measured and recorded [97,98].

The minimum inhibitory concentration (MIC) of the ZnO-NP nanofluid was estimated with the agar well diffusion method previously mentioned, using concentrations starting from 1000 µg/mL and diluted double-fold to 7.8 µg/mL. After incubation, the lowest concentration causing an inhibition zone could be determined as the MIC [99]. The experiment was repeated twice to confirm the obtained results.

#### 4.6.2. Determination of ZnO-NPs’ Nematicidal Activity by Mortality Assay of *Meloidogyne incognita*

*Meloidogyne incognita* pure culture was originated from the single egg mass of an identified *M. incognita* female. The soil surrounding the root of a tomato infected for 45 days was used as a source of root-knot nematodes (RKNs). The infective second-stage juveniles (J_2_s) of *M. incognita* were isolated using the sieving and decanting procedure [100]. The nematicidal activity of ZnO-NP nanofluid against *M. incognita* J_2_s was tested in vitro using a mortality assay after 24, 48 and 72 h of exposure. In sterile test tubes, a stock solution of ZnO-NP nanofluid was diluted with sterile distilled water to reach a final concentration of 100 µg/mL of ZnO-NPs and a final volume of 5 mL, with 100 ± 5 J_2_s of *M. incognita.* In addition, a treatment that contained the same volume of base nanofluid without the ZnO-NPs and an autogenic control that contained only sterile distilled water, both including 100 ± 5 J_2_s of *M. incognita*, were also tested. Treatments and controls were performed in ten replicates and all tubes were maintained in an incubator at 30 °C. The count of live and mortal juveniles after each time interval was estimated using a one mL counting slide. The mobility of nematodes or their winding shape indicated their vitality, while immobility indicated mortality. The mortality percentage was estimated as [(number of live nematode larvae in control treatment) − (number of live nematode larvae counted in other treatments)/(number of live nematode larvae in control treatment)] × 100.

### 4.7. Impact of ZnO-NP Nanofluid on Seed Germination Seedling Vigor Using V. faba Plant Model

To ensure surface sterility, *V. faba* seeds that were healthy and uniform were immersed in 5% NaOCl for 10 min and washed three times with distilled water. At 25 °C, the seeds were germinated on filter sheets saturated with the tested concentrations of ZnO-NPs (12.5, 25, 50, 100 and 200 µg/mL). The same dilutions of base fluid without ZnO-NPs served as the vehicle control and distilled water served as a negative control. Each treatment had three replicates of ten seeds per dish. After 5 days of treatment, the numbers of seedlings were counted as a measure of the germination percentage, with a 2 mm radicle emerging from the seeds as they germinated. Regular measurements of shoot height and root length were taken until the 12th day [101].

### 4.8. Influence of ZnO-NPs on Cytological Characteristics of V. faba

*V. faba* seeds were grown on filter papers saturated with distilled water until the root attained a length of around 1 cm. Roots were subjected to the previously mentioned concentrations for 2, 4 and 6 h. Three replicates were performed for each case. The roots were preserved in a 3:1 ethanol:acetic acid (Adwa, Cairo, Egypt) solution for 24 h before being hydrolyzed with 1 N HCl in a water bath at 60 °C for 10 min. The Feulgen squash technique was then used to dye the root tips [102]. Using an optical microscope at a magnification of 40, a minimum of 2000 cells from the control and from all treatment classes were scored to measure the mitotic index and chromosomal abnormalities.

The ratio of the total number of dividing cells to the total number of cells counted × 100 was used to obtain the percentage of the mitotic index. The ratio of the number of cells at each phase to the total number of dividing cells observed × 100 was used to obtain the percentage of the phase index. The total chromosomal abnormality (CA) percentage (the number of cells with chromosomal alterations/number of cells in division × 100) was calculated by counting different stages of mitosis. We counted micronuclei (MNs) with a diameter of less than one-third of the main nucleus. The number of cells with micronuclei per 1000 scored interphase cells was used to calculate the frequency of MNs [68].

### 4.9. Statistical Analysis

The resulting data were submitted to analysis of variance (ANOVA) and compared with the Tukey test with a significance level of 5% using MINITAB statistical software version 18.1 (MINITAB Inc., State College, PA, USA).

## 5. Conclusions

Biosynthesized ZnO-NPs are believed to have promising efficacy as growth enhancers in addition to their antimicrobial activity. In this study, a strain of actinomycetes capable of forming ZnO-NPs was isolated and identified as the *Streptomyces plicatus* MK-104 strain using several identification approaches. According to the results of several characterization techniques, the ZnO-NPs were produced and appeared spherical, with average sizes ranging from 21.72 to 22.4 nm. ZnO-NPs were shown to be effective as an antagonist for various plant pathogens, such as bacteria, fungi and nematodes. Furthermore, when moderate concentrations were employed, they enhanced seed germination, increased shoot and root lengths and demonstrated no significant toxicity against the *Vicia faba* plant in vitro. Therefore, biosynthesized ZnO-NPs could be utilized as a suitable alternative for the control of numerous plant pests, as well as to enhance plant growth. However, confirmation of this may need further research.

## Figures and Tables

**Table 1:** MK-104 isolate culture properties on various ISP media.

Medium	Growth	Aerial Mycelium	Substrate Mycelium	Diffusible Pigment
ISP-1	Good	White	Yellow	No
ISP-2	Good	Grey	Light brown	No
ISP-3	Very good	Grey	Brown	No
ISP-4	Very good	Grey	Brown	No
ISP-5	Good	Hygroscopic growth (without sporulation)	Yellow	No
ISP-6	Good	Hygroscopic growth (without sporulation)	Yellow	No
ISP-7	Very good	Light grey	Light brown	No

**Table 2:** Physiological characteristics of isolate MK-104.

Utilization of Carbon Sources	Results	NaCl Tolerance (%)	Results
D-Glucose	++	0	+
L-Rhamnose	++	1	++
D-Xylose	++	2	++
D-Mannitol	++	3	++
Inositol	++	4	++
Sucrose	+	5	++
L-Arabinose	++	6	+
Fructose	++	7	+
Cellulose	+	8	−
Growth at different temperatures (°C)		Utilization of nitrogen sources	
10	−	L-Asparagine	+
20	+	L-Cysteine	−
30	++	L-Valine	+
40	++	L-Threonine	+
50	+	L-Phenylalanine	++
60	−	L-Methionine	+
Growth at different pH values		L-Histidine	++
4	−	L-Arginine	++
5	+	Tolerance to toxic substances	
6	+	Sodium azide 0.01% (*w/v*)	−
7	++	Phenol 0.1% (*w/v*)	−
8	++	Crystal violet 0.001% (*w/v*)	±
9	+	Growth on Czapek’s medium	±
10	−		

±, positive; −, negative; +, moderate growth; ++, good growth.

**Table 3:** Biochemical characteristics of isolate MK-104.

No.	Biochemical Tests	Results
1	Lipid hydrolysis	+
2	Starch hydrolysis	+
3	Gelatin hydrolysis	+
4	Casein hydrolysis	−
5	Tyrosine degradation	+
6	Urea degradation	+
7	Pectin degradation	+
8	Esculin degradation	+
9	Citrate utilization	+
10	Motility test	−
11	H2S production	−
12	Lecithin degradation	+

+, positive; −, negative.

**Table 4:** Antimicrobial activity and MIC values of biosynthesized ZnO-NPs.

No.	Microorganisms	Mean Diameter of Inhibition Zone (mm) ± Std. Error			MIC (µg/mL)
		ZnO-NP Nanofluid	Base Fluid	Control AB	
1	*Erwinia amylovora*	26.6 ± 0.667	0	SXT 19.6 ± 0.667	15.6
2	*Aspergillus flavus*	17.6 ± 0.667	0	FLU 0	250
3	*Aspergillus niger*	15	0	FLU 0	125
4	*Fusarium oxysporum*	21 ± 0.577	0	FLU 0	62.5
5	*Fusarium moniliform*	22 ± 0.577	0	FLU 17.6 ± 0.882	250
6	*Alternaria alternata*	17 ± 0.667	0	FLU 0	500
7	*Aspergillus oryzae*	18.3 ± 0.882	0	FLU 0	250
8	*Aspergillus fumigatus*	17.3 ± 0.667	0	FLU 0	250

**Table 5:** Exposure to ZnO-NPs at different concentrations and for different durations of time affected the mitotic index and phase index.

Treatments	Time	Mitotic Index	Prophase Index	Metaphase Index	Ana Phase Index	Telophase Index
D.W	2	14.93 ± 0.9	37.55 ± 2.9	28.18 ± 4.67	19.90 ± 3.6	4.36 ± 4.2
	4	14.93 ± 0.9				
	6	14.93 ± 0.9				
Base 12.5	2	18.53 ± 4.0	45.44 ± 3.0	33.30 ± 3.33	13.50 ± 3.5	7.750 ± 1.87
	4	14.7 ± 2.8				
	6	11.6 ± 1.2				
12.5 µg/mL	2	28.73 ± 3.0	48.42 ± 3.0	26.61 ± 5.53	15.12 ± 5.07	9.82 ± 2.31
	4	20.06 ± 1.0				
	6	13.66 ± 1.0				
Base 25	2	16.86 ± 1.0	50.68 ± 4.0	26.21 ± 3.9	15.68 ± 3.7	7.42 ± 2.59
	4	14.6 ± 1.9				
	6	7 ± 2				
25 µg/mL	2	17.76 ± 1.0	45.31 ± 6.5	27.88 ± 7.5	12.09 ± 2.5	14.70 ± 2.03
	4	14.7 ± 1.5				
	6	7.36 ± 1.5				
Base 50	2	17.4 ± 2.8	38.69 ± 3.5	31.81 ± 2.8	11.46 ± 2.3	18.02 ± 1.81
	4	14.5 ± 1,8				
	6	5.2 ± 0.55				
50 µg/mL	2	17 ± 1.4	41.62 ± 7.9	24.45 ± 2.9	10.81 ± 4.97	23.09 ± 4.54
	4	17.36 ± 1.0				
	6	11.26 ± 2.0				
Base 100	2	4.8 ± 1.01	0 ± 0	66.55 ± 2.8	25.20 ± 3.53	8.24 ± 0.73
	4	3 ± 0.90				
	6	2.8 ± 1.01				
100 µg/mL	2	7.93 ± 0.5	15.65 ± 5.8	34.02 ± 4.1	36.06 ± 5.17	14.26 ± 4.35
	4	4.93 ± 1.7				
	6	2.9 ± 0.85				
Base 200	2	2.93 ± 0.6	0 ± 0	42.56 ± 9.1	25.01 ± 5.13	32.41 ± 10.3
	4	2 ± 0.55				
	6	2.1 ± 0.36				
200 µg/mL	2	5 ± 0.60	0 ± 0	22.01 ± 5.45	35.09 ± 8.69	42.88 ± 6.87
	4	4.3 ± 1.3				
	6	2.5 ± 0.7				

## Data Availability

The study did not report any data. All data, tables and figures in this manuscript are original.
